# Are Bank Employees Stressed? Job Perception and Positivity in the Banking Sector: An Italian Observational Study

**DOI:** 10.3390/ijerph15040707

**Published:** 2018-04-10

**Authors:** Alice Mannocci, Laura Marchini, Alfredo Scognamiglio, Alessandra Sinopoli, Simone De Sio, Sabina Sernia, Giuseppe La Torre

**Affiliations:** 1 Department of Public Health and Infectious Diseases, Sapienza University of Rome, Piazzale Aldo Moro 5, 00185 Rome, Italy; alessandra.sinopoli@uniroma1.it (A.S.); giuseppe.latorre@uniroma1.it (G.L.T.); 2 Statistical Unit, Trade Union Fisac-CGIL, 56100 Pisa, Italy; laura_marchini@hotmail.com; 3 National Department of Health and Safety, Trade Union Fisac-CGIL, 00100 Rome, Italy; fredsco@libero.it; 4 Research Unit of Occupational Medicine, Sapienza University of Rome, 00185 Rome, Italy; simone.desio@uniroma1.it; 5 Center of Occupational Medicine, Department of Public Health and Infectious Diseases, Sapienza University of Rome, 00185 Rome, Italy; sabina.sernia@uniroma1.it

**Keywords:** stress, job demand, decision latitude, positivity, banking employees, bank

## Abstract

*Background*: The epidemiology of stress on bank workers in Europe is only at the introductory stages. This study investigated for the first time the association between occupational stress level in bank-employees using the BEST8, Karasek-Model and socio-demographic and working factors in Italy. *Methods*: The observational pilot study involved 384 employees. Three questionnaires were adopted to collect data: Karasek-Model, BEST8 (*p* < 0.001) and Positivity-Scale. *Results*: 25% of the sample belonged to high stress group. The workers more stressed were older with a commercial role and consumer of antidepressants/sedatives. Women were much more likely to agree with the perception of feeling unsafe in a possible robbery (OR = 2.42; 95% CI: 1.50–3.91) and with that sales requests were in conflict with one’s own personal moral code (OR = 2.31; 95% CI: 1.38–3.87). Older employees declared feeling inadequate in the workplace (OR = 1.97; 95% CI: 1.07–3.65) and younger employees referred to be anxious about meeting financial budget goals. Workers who had a low positivity had a lower probability of adaptation (OR = 0.88; 95% CI: 0.83–0.93). *Conclusions*: The occupational stress level in the banking sector involves many aspects: gender, type of bank, role, personal morals, high job-demands, low level of decision-making. This study recommended that banks should implement strategic interventions for well-being of employees, and consequently for their productivity.

## 1. Introduction

Globalization and the new economy have determined significant changes in the organization and management of work. The banking sector presents several symptoms of this evolution: mass layoffs, acquisitions and crashes, digitalization, outsourcing, business re-engineering with the reduction of hierarchical levels, job insecurity, increasing competition due to the entrance of more private banks (corporate), and multifunctional tasks. Recent literature underlines that these symptoms determine high levels of stress in bank employees [1,2,3].

According to the World Health Organization (WHO), occupational stress is “the response people may have when presented with work demands and pressures that are not matched to their knowledge and abilities and which challenge their ability to cope” [4]. It is well known that the exposure to stress, especially prolonged stress, can be associated to poor health (mental and physical disorders), unhealthy habits (alcoholism, drug addiction), absenteeism, reduced efficiency (indifference, apathy, lack of motivation or creativity), and even death [5,6]. The banking sector presents other peculiar aspects that could be considered potential sources of stress for employees: possible violence, risk of robbery, as well as conflicting tasks, pressure to achieve business targets, and geographical transfer/mobility also [7].

In recent years, there have been many activities created to promote the management and assessment of occupational stress, and in Italy too [8,9,10], but findings haven’t been applied in all realities and in standardized ways. 

There is a conspicuous body of literature on stress disorder among bank employee victims of robbery at the European level [11,12,13,14]. These studies substantially agree that the several peri-trauma factors and post- trauma variables can increase the risk of workers developing post-traumatic stress reaction.

On the other hand, few publications have explored the level of stress and depression in bank workers, and whether this is related to socio-demographic characteristics and/or working environment at the European level. Michailidis and Georgiou [15] studied the degree of occupational stress for different groups of bank workers in Cyprus reporting that a high academic degree level of qualification was associated with feeling more satisfied with their job, and with their achievement in the organization. In addition, a positive and ambitious attitude to living allows employees to have more time to relax, thus reducing their levels of stress to a minimum. Amigo et al. showed that Spanish bank staff had high levels of burnout syndrome (BS) and that emotional exhaustion was the main factor. There was a greater risk of burnout for those working in branch offices than for those in central services and of a close correlation between burnout and interpersonal stress at work on a daily basis because of the commercial strategies the sector has used in recent years [16]. Another European study conducted by Seegers and van Elderen investigated how stressors related to work affected the physical and psychological wellbeing in a large Dutch banking organization, and what levels of absenteeism they gave rise to. They found that subjective stressors and stressors related to work did well as predictors of psychological strains and complaints, which may become health problems for the affected employee, but they were inadequate as a way to explain absenteeism [17].

In 2017 Mannocci et al. [18] published a set of key questions focused on the aspects potentially related to the stress among bank employees and assessed their reliability. This tool consists of eight items and is called Bank Employee Stress Test 8 (BEST8).

In order to assess the possible association with the BEST8 items with the stress level, the present study hypothesizes the relationship between the items of BEST8 with the validated Job Demand Control Model (JDCM). The reason for this choice is the fact that this model is one of the most widely studied models for occupational stress [19]. Furthermore, given the reduced scientific production, and the availability of the new tool, the following aims have been also performed: to increase the knowledge on the occupational stress level in workers of banking sector in Italy, and to evaluate the possible association of the BEST8 items with socio-demographic characteristics and working/job conditions and the two basic dimensions stress level of JDCM (job demands and decision latitude).

## 2. Material and Methods

### 2.1. Study Design and Setting

This cross-sectional study was conducted in all banks (N = 10 banks, both national and local) located in the district area of Pisa, Tuscany, Italy, between April and November 2016. The study included all employees independently from their job positions and job seniority. An on-line anonymous questionnaire was self-administered to establish baseline characteristics and risk factors. Four sections were included:

1. Socio-demographic aspects and work/job characteristics;
2. BEST8: a questionnaire on occupational stress among bank employees [18];
3. Job Content Questionnaire (JCQ) [20] in order to perform the JDCM;
4. Positivity Scale [21].

The following socio-demographic variables were collected: gender, age, marital status, if they had children, smoking habits, previous or present consumption of antidepressants or sedatives (drugs). The work characteristics included were the following: type of bank (national or local), job position (employee or manager), commercial role (yes/no), and type of job contract (indeterminate vs. fixed term).

The administered BEST8 questionnaire was designed ad hoc for banking sector workers and estimates their stress condition: it includes eight items with two possible answers (I agree/I don’t agree).

Furthermore, the short version of the JCQ was used, and it was developed following the JDCM. This version comprises 15 items with four answer options arranged in a Likert-type scale, ranging between “strongly agree” and “strongly disagree”. Groups of these questions were combined to define the “Job Demand” and “Decision Latitude” (or “Job Control”) scores.

Finally the Positivity Scale, composed of a set of eight items assessing an individual’s positive attitude about himself, about his life, and his attitude towards the future was also administered. The final score ranges from 8 to 40: each item was formatted with 5-point Likert scales ranging from 1 (strongly disagree) to 5 (strongly agree).

The online questionnaire was created using Google Forms and, at the end of the data collection phase, the information was imported from Google Doc into an Excel spreadsheet. Data were collected between April and October 2016. The questionnaire was administered in an anonymous way.

All bank employees were invited to fill in the questionnaire by Federazione Italiana Sindacale Assicurazioni e Credito (FISAC) trade union. The FISAC provided us a list of 414 employees who had a registered email address. The mailing list was used to contact the workers. Furthermore, one worker within each bank (with a total of 10 bank branches being involved) was chosen as a supporter of the study in order to solicit all colleagues to fill in the questionnaire during the period of administration. The ethical approval of the study protocol was obtained from the local Ethics Committee (prot. 27/17 RIF.CE 4268). Sample size calculations were based on computation of single group mean. The following parameters were considered for the estimate:

- power 80%;
- significance level 95%;
- size of the target population N = 2000 (workers in the Banking sector from the Pisa District) [22];
- the reference mean value of stress level in the administrative sector: the mean of Job demand is 67 (SD = 18.3) [23]);
- a worst value of ±5: Job Demand ranged 67 ± 5.

The sample size obtained was N = 322. The size was increased by 10% in order to offset the effect of potential missing data. The final sample size was N = 350.

### 2.2. Statistical Analyses

The statistical analysis was performed considering the following steps:

- description of the demographic-job characteristics and the positive outlook of the sample;
- description of the working context perception (BEST8′s items and demand-control scores);
- univariate analysis in order to assess the differences in workplace context perception by gender, age, job characteristics, occupational stress level and positive outlook;
- a post hoc chi-square analysis, using the standardized residual for each cell can be used to determine which discrepancies between observed and expected values are larger than might be expected by chance [24];
- eight logistic regression models were computed, estimating OR with 95% confidence intervals (95% CIs): the dependent variable in the models was each item of the BEST8 tool, and the independent variables were the demographic-job characteristics, job demand, decision latitude and positivity scale;
- analysis of JDCM was performed using tables and scatter-plots, stratifying by gender, age group, type of job, commercial role, type of bank.

The ordinal variables with Likert-points answers were converted into dichotomous variables, according to MacCallum et al. [25]. This allowed us to use in a logistic regression model the dependent variable as a dichotomous one. The statistical significance was set at *p* < 0.05. The software used to analyze data was SPSS 23 for Windows (SPSS Inc., Chicago, IL, USA).

## 3. Results

A total of ten bank branches were involved in this study and the total potential eligible participants was 625. Of these workers, 414 were contacted by email, since they were “active contacts” from employee’s databases. Other 106 workers were contacted with the personal communication by the responsible of the study. Therefore, a total of 520 workers were approached to participate in the study. Three hundred and eighty-four questionnaires were collected (total response rate = 74%). Of these, two were only partially answered (0.5%) and were not included in the inferential statistical analysis. The demographic-job characteristics of the sample are described in Table 1. Fifty-five percent of the sample was female; 29% of respondents were aged less than 44 years and 53% were aged between 45 and 55; about 40% of employees worked in a local bank and 70% had a commercial role.

**Figure 1:**
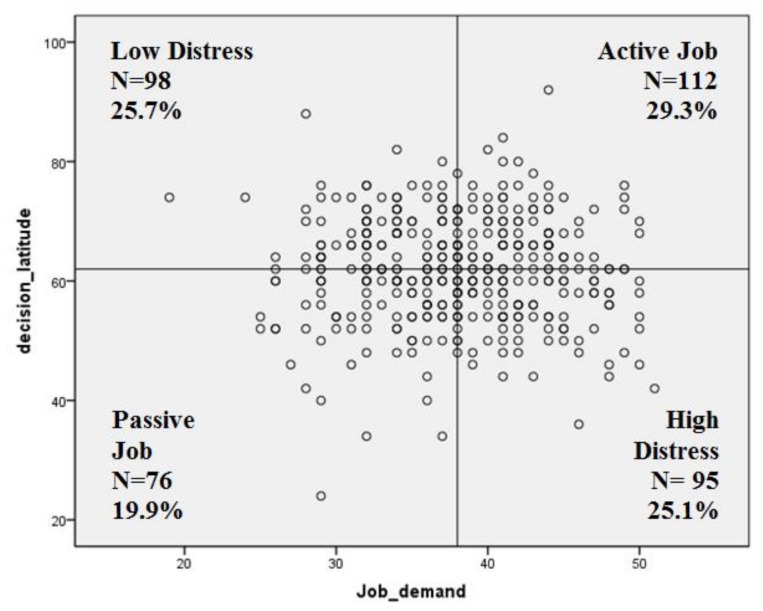
Scatter-plot of Job Demand-Control Model (sample N = 382).

The median Job Demand was 38 (25th percentile = 34; 75th percentile = 42), whereas the median value of the decision latitude was 62 (25th percentile = 56; 75th percentile = 68). Figure 1 shows the workers’ individual values in the JDCM. About 30% of the respondents were in the Active Job quadrant and 25% in the high distress quadrant. Two workers did not fill in this part of the questionnaire (missing).

The differences in JDCM within different categories are described in Table 2. There were not any significant different distributions when stratifying by gender, whether employees had children, and whether they were daily smokers (*p* > 0.05). On the other hand, there were significant associations with all followed variables: age, anti-depressant consumption, role, type of bank and job position. In particular in the group of passive jobs there are more older workers than younger workers (36% versus 17% and 15%) and there are workers without commercial role than with commercial role (30% versus 15%). In addition, those who used drugs had a higher significant percentage to stay in high distress category (38% versus 20%). The class of workers in local banks, compared to national one, was associated to the low distress group (35% versus 20%). Finally, in the group of active job there are more managers than employees (43% versus 25%).

### BEST8 Test Results

Table 1 reports the percentage of agreement with the BEST8 items. A high overall percentage (over 65%) of agreement was registered in the sample, by item. The agreement was as follows: recommending bank products occurred just because this task was in the budget (83.4%); anxiety due to possible failure to achieve the budget targets (82.4%); stress for frequent company re-organization (77.9%); the risk of robbery was considered as an uncomfortable condition (75.1%); the sales requests were in conflict with one’s own consideration of what is morally right (68.5%).

Concerning the univariate analysis of the BEST8 (Table 2), seven items had a significant association with the JDCMs classification; only one item, regarding the possibility of robbery, was not significantly associated to the Model (*p* = 0.177). A higher percentage of distress was present in the following situations (*p* < 0.05): “The failure to achieve the budget targets causes me anxiety”, “I’m not comfortable recommending a bank product just because it is in the budget”, “The sales requests and/or consultations are in conflict with what I consider to be morally right”, “My colleagues or superiors ask me to be more flexible with my job”.

Finally, those who did agree with the item “I have time to dedicate myself to my hobbies/activities/stuff” had a higher percentage of low stress (34% versus 19%) and no active job (36% versus 21%).

The univariate analysis of the BEST8′ items versus gender, age, having children, and job characteristics is reported in supplementary tables.

Table 3 and Table 4 show eight logistic regression models considering the dependent variable as each of the BEST-8 items. Some significant associations seen in the univariate analysis did not remain significant after adjustment for age, sex, and other established factors. The significant results of the models are described below. 

Women were more likely than men to agree with all items of the BEST8 (*p* < 0.001), but the items “The frequent Company re-organization makes me feel uncomfortable” and “I have time to dedicate myself to my hobbies/activities” were the exceptions: the first item was not significant whilst the second indicates that females were more likely than men to disagree with this.

As regards age, the model shows a significant association between being older and risk of considering workplace change as exceeding their capacity for adaptation (Table 3), OR = 1.97 with 95% CI: 1.07–3.65. The younger group presented a significant double risk of having anxiety for failure to achieve budget targets, and the possibility of a ‘mobility procedure’, compared to the 45–54 age group, OR = 0.50 with 95% CI: 0.27–0.91 (Table 3). 

Having children was associated with having no time for themselves (OR = 0.48; 95% CI: 0.29–0.79).

As regards the type of bank, National bank workers had a significantly higher risk of having anxiety for failure to achieve budget targets, and a greater perceived possibility of incurring in a ‘mobility procedure’ (or geographical transfer) (OR = 1.93; 95% CI: 1.07–3.48), of considering that change in the workplace exceeded their capacity for adaptation (OR = 1.85; 95% CI: 1.17–2.93), of having conflict with what they considered as morally right (OR = 4.24; 95% CI: 2.45–7.34) and for having time to dedicate themselves to hobbies/activities (OR = 1.97; 95% CI: 1.22–3.18).

The commercial role and job position were not significantly associated to occupational stress items among bank employees.

As regards tobacco addiction, employees who smoked had time to dedicate to themselves (OR = 1.80; 95% CI: 1.01–3.20).

Workers with previous or present drug consumption had a higher likelihood of having anxiety for failure to achieve the budget targets, and a higher likelihood of perceiving the possibility of a ‘mobility procedure’ (OR = 3.77; 95% CI: 1.51–9.42) and of considering change in the workplace as exceeding their capacity for adaptation (OR = 2.0; 95% CI: 1.16–3.46).

There were associations between decreasing scores on the positivity scale as a continuous variable, and anxiety for failure to achieve the budget targets (OR = 0.88; 95% CI: 0.83–0.93), low confidence in one’s own capacity for adaptation (OR = 0.92; 95% CI: 0.85–0.99), and having conflict with what they considered as morally right (OR = 0.90; 95% CI: 0.84–0.94). On the contrary, there was an association between increasing scores on the positivity scale and having time for activities/hobbies (OR = 1.06; 95% CI: 1.01–1.13).

Increasing Job Demand scores were significantly (*p* < 0.05) associated with all items of the BEST-8. Only “Feelings of fear for a possible bank robbery” was an exception (*p* ≥ 0.05).

The Decision Latitude score was significantly associated with following items: “The failure to achieve budget targets causes me anxiety” (OR = 0.96; 95% CI: 0.93–0.99); “I’m not comfortable recommending a bank product just because it is in the budget”(OR = 0.95; 95% CI: 0.92–0.99); “Frequent Company re-organization makes me feel uncomfortable” (OR = 0.95; 95% CI: 0.92–0.98); “the sales requests are in conflict with what I consider as morally right” (OR = 0.94; 95% CI: 0.91–0.97); “my colleagues or superiors ask me to be more flexible with my job” (OR = 0.95; 95% CI: 0.93–0.98).

## 4. Discussion

The object of the present study was to evaluate the stress level in a pilot sample of bank employees in Italy. The results show that 82% of bank workers are anxious about the failure to reach set goals, 84% of respondents declared that they were uncomfortable recommending customers a product just because it was in their budget goal, 64% of respondents said that the pressure to sell was in conflict with what they believed to be as morally correct, and 63% declared that they had been urged by their superior to be “elastic” in performing their duties.

All items of the BEST8 resulted associate with the JDCM at significant level (the first item on risk of robbery is an exception) and this confirm that the BEST8 is a tool that adapts to the measurement of stress and vice versa of the bank employees. Finally, about one out three of respondents reported to have used tranquilizers, sedatives or antidepressants.

The literature regarding the assessment of the demand control model in the banking sector reveals that this topic has not been adequately investigated. The present study found that 25% of respondents were in the high stress area (distress area) of the JDCM. The literature highlights the need to intensify preventive efforts that might modify the stress threat among these workers [26].

The main socio-demographic factors associated to high levels of occupational stress, as measured with BEST8, were gender, years of employment and commercial role. The same significant associations were confirmed when considering the JDCM, though only gender in this case was the exception (*p* = 0.145).

This no significant association with gender in the JDCM is in line with several scientific articles [26,27,28]. On the other hand, the BEST8′s items underlined significant differences by gender: women suffered the working conditions of the current organizational model, and had double the risk of using drugs than men. A possible explanation could be that the BEST8 was used as a tool ad-hoc for the bank sector. There are not publications that use this tool for testing the association between gender and stress level in the banking sector. Unfavorable work conditions for women have also been highlighted in recent publications that show how it is more difficult for women to get top jobs or roles in banks [29,30].

In the banking sector, the requirements of work need continuous improvement and upgrading of skills as well as training to keep up with new forms of work organization, and this factor could be a further source of stress [31].

Finally, the use of positivity scales in the banking sector has still not been studied in the scientific literature. This investigation represents the first application of this tool, and comparisons are not possible. However, based on the findings of this study, the positivity scale was associated to issues concerning the capacity of worker adaptation, and with having time for themselves, thus resulting in a low level of stress. This finding is in line with findings of previous research on this topic: Hausmann et al. [32], Michailidis and Georgiou [15] have stated that both cultural positivity and life satisfaction can help in allowing bank employees to reduce their levels of stress, and positivity also appears to be linked with high levels of academic qualifications.

The strengths of the present study include an adequate sample size from a homogeneous population of employees of Tuscany. In addition, levels of stress were quantified with validated questionnaires that are currently available. The low number of missing values in the answers didn’t change the research findings.

There were several limitations of this study. Firstly, the results could be biased due to the self-reported survey format used. For example, data on smoking status and drug use were self-reported, and underreporting may have occurred. Secondly, since this research was based on a cross-sectional study, it was not possible to examine a possible cause-and-effect relationship. In fact, there is a need for caution in how the results related to drug use are presented: a cross-sectional study of this nature cannot make any pronouncement on causes and effects, so there is no way of knowing whether or not to take care of the anxieties caused by their job—or have (unrelated) anxiety problems which may make them more likely to feel anxious or worried about work factors as a result. 

Furthermore, in view of the complexity of the outcome and the subjective perception of stress, the survey did not include possible confounding variables such as clinical, psychological and somatic factors, or length of employment at the bank and other aspects such as, for example, branch size (how many colleagues work with the respondent). Moreover, a possible limitation of the analysis derives from the transformation of variables with Likert-points answer into dichotomous ones. However, even if there is not a complete agreement on this point, we followed the procedure described by McCallum et al. [25]. Finally the sample could be not representative of the population of Italian Bank employees, because only one district was been involved.

## 5. Conclusions

In conclusion, the health and safety risks have usually been considered as marginal in the banking sector, but in recent years we have seen a worsening of job context due to frequent reorganization of the business model, revolution and digitization, and the pressure to sell [33]. The present study clearly shows the consequences of this trend on employee stress, and highlights the need for interventions to prevent stress disorders among bank workers. The study also identifies categories for which the risks are greater: women, drug consumers and some age groups. The findings of the present research represent an input for bank workplace management as distress has been shown to have a detrimental effect on the health and wellbeing of bank employees, as well as a negative impact on an employee’s performance, and consequently on workplace productivity and profits [34,35,36].

## Figures and Tables

**Table 1:** Characteristics of the sample.

Qualitative Variables		N (%)
Gender	Male	174 (45.3)
	Female	210 (54.7)
Age (years)	≤44	170 (29.4)
	45–55	201 (52.4)
	Over 55	70 (18.2)
Civil status	Single	52 (13.5)
	Married/domestic partnership	296 (77.1)
	Separated/divorced	32 (8.3)
	Widowed	4 (1.0)
Sons	Yes	271 (70.6)
	No	113 (29.4)
Daily Smoker	No	306 (79.7)
	Yes	78 (20.3)
Antidepressants or sedatives drugs ^a^	No	276 (71.9)
	Yes	108 (28.1)
Bank	Local	148 (38.5)
	National	236 (61.5)
Job position	Employee	286 (74.5)
	Manager	98 (25.5)
Job contracts	Indeterminate	372 (97)
	Fixed term	11 (3.0)
Commercial role	No	115 (30.0)
	Yes	269 (70.0)
**BEST8 Questionnaire**		
1-In terms of safety, It makes me uncomfortable thinking about a possible robbery on my desk.	I agree	289 (75.1)
	I don’t agree	95 (24.9)
2-The failure to achieve the budgets targets causes me anxiety, because there are risks of geographical mobility and/or of the switch of duties.	I agree	315 (82.4)
	I don’t agree	69 (17.6)
3-The pace of change on work place exceeds my capacity for adaptation.	I agree	225 (58.5)
	I don’t agree	159 (41.5)
4-I’m not comfortable recommending a bank product just because in the budget.	I agree	322 (83.4)
	I don’t agree	62 (16.6)
5-Frequent Company’s re-organization makes me feel uncomfortable.	I agree	300 (77.9)
	I don’t agree	84 (22.1)
6-The requests of sales and/or consultations are in conflict with what I consider morally right.	Yes	123 (68.5)
	No	242 (31.5)
7-I have time to dedicate myself to my hobbies/activities/stuff.	Yes	175 (46.1)
	No	206 (53.9)
8-My colleagues or superiors ask me to be more flexible with the job.	Yes	242 (63.6)
	No	139 (36.4)
**Quantitative variables**	**Mean (SD)**	
Job Demand	37.9 (5.8)	
Decision Latitude	62.0 (9.0)	
Positivity Scale	24.0 (4.2)	

^a^ Previous or present consumptions of antidepressants or sedatives drugs.

**Table 2:** Association between JDCM and demographic-job characteristics of the employees.

		Job Demand-Control Model					
		Active Job	Low Distress	Passive Job	High Distress		
		N (%)	N (%)	N (%)	N (%)	Tot	*p* ^b^
**Qualitative Characteristic**							
Gender	Male	51 (29)	42 (24)	43 (25)	38 (22)	174	0.145
	Female	61 (29)	56 (27)	33 (16)	58 (28)	208	
Age (years)	≤44	50 (30)	44 (26)	29 (17)	46 (27)	169	0.020
	45–55	47 (33)	40 (28)	22 (15)	35 (24)	144	
	Over 55	15 (22)	14 (20)	25 (36) ^e^	15 (22)	69	
Civil status	Single	16 (31)	14 (27)	10 (19)	12 (23)	52	^c^
	Married/domestic partnership	91 (31)	70 (24)	58 (20)	76 (26)	295	
	Separated/divorce	4 (13)	13 (42)	7 (23)	7 (23)	31	
	Widowed	1 (25)	1 (25)	1 (25)	1 (25)	0	
Children	Yes	83 (31)	69 (26)	57 (21)	61 (23)	270	0.298
	No	29 (26)	29 (26)	19 (17)	35 (31)	112	
Daily Smoker	No	87 (29)	80 (26)	66 (22)	71 (23)	304	0.184
	Yes	25 (32)	18 (23)	10 (13)	25 (32)	78	
Antidepressants or sedatives (drugs) ^a^	No	79 (29)	83 (30) ^d^	58 (21)	56 (20)^d^	276	0.001
	Yes	33 (31)	15 (14) ^d^	18 (17)	40 (38)^d^	106	
Bank	Local	40 (27)	52 (35) ^d^	29 (20)	26 (18)	147	0.002
	National	72 (31)	46 (20) ^d^	47 (20)	70 (30)	235	
Job position	Employee	70 (25) ^d^	77 (27)	58 (20)	79 (28)	284	0.006
	Manager	42 (43) ^d^	21 (21)	18 (18)	17 (17)	98	
Commercial role	No	23 (20)	34 (30)	35 (30) ^d^	23 (20)	115	0.001
	Yes	89 (33)	64 (24)	41 (15) ^d^	73 (27)	267	
**BEST8 Questionnaire**							
1. In terms of safety, It makes me uncomfortable thinking about a possible robbery at my desk.	I agree	83 (29)	76 (26)	51 (18)	78 (27)	288	0.177
	I don’t agree	29 (31)	22 (23)	25 (27)	18 (19)	94	
2. The failure to achieve the budget targets causes me anxiety, because there are risks of geographical transfer (mobility) and/or of a switch of duties.	I agree	98 (31)	67 (21) ^d^	59 (19)	90 (29) ^d^	314	<0.001
	I don’t agree	14 (21)	31 (46) ^d^	17 (25)	6 (9) ^d^	68	
3. The pace of change in the workplace exceeds my capacity for adaptation.	I agree	73 (33)	42 (19) ^d^	41 (18)	68 (30)	224	<0.001
	I don’t agree	39 (25)	56 (35) ^d^	35 (22)	28 (18)	158	
4. I’m not comfortable recommending a bank product just because it is in the budget.	I agree	96 (30)	70 (22) ^d^	64 (20)	91 (28) ^d^	321	<0.001
	I don’t agree	16 (26)	28 (46) ^d^	12 (20)	5 (8) ^d^	61	
5. Frequent Company re-organization makes me feel uncomfortable.	I agree	96 (32)	59 (20) ^d^	61 (20) ^d^	83 (28)	299	<0.001
	I don’t agree	16 (19)	39 (47) ^d^	15 (18) ^d^	13 (16)	83	
6. The sales requests and/or consultations are in conflict with what I consider to be morally right.	Yes	67 (28)	39 (16) ^d^	53 (22)	82 (34) ^d^	241	<0.001
	No	40 (33)	49 (40) ^d^	20 (16)	13 (11) ^d^	122	
7. I have time to dedicate myself to hobbies/activities/stuff.	Yes	36 (21) ^d^	59 (34) ^d^	45 (26)	34 (19)	174	<0.001
	No	74 (36) ^d^	39 (19) ^d^	30 (15)	62 (30)	205	
8. My colleagues or superiors ask me to be more flexible with the job.	Yes	77 (32)	42 (17) ^d^	48 (20)	75 (31) ^d^	242	<0.001
	No	35 (26)	55 (40) ^d^	27 (20)	20 (15) ^d^	137	

^a^ Previous or present consumption of antidepressants or sedatives (drugs); ^b^ the *p*-value of the χ^2^ test; ^c^ the computation is not possible: in the χ-square test 4 cells (25%) have an expected count of less than 5, and the minimum expected count is 0.80; ^d^ the d indicates statistical significance at the adjusted α level of 0.006 applying the post-hoc test of chi-square test; ^e^ the e indicates statistical significance at the adjusted α level of 0.004 applying the post-hoc test of chi-square test.

**Table 3:** Multivariate logistic regression models concerning the first four BEST8′ items.

Covariates		In Terms of Safety, It Makes Me Uncomfortable Thinking about a Possible Robbery on My Desk		The Failure to Achieve the Budget Targets Causes Me Anxiety, Because There Are Risks of Geographical Mobility and/or of the Switch of Duties		The Pace of Change on Work Place Exceeds My Capacity for Adaptation		I’m Not Comfortable Recommending a Bank Product Just Because in the Budget	
		OR	95% CI	OR	95% CI	OR	95% CI	OR	95% CI
**Gender**	**Male ^b^**	1		1		1		1	
	**Female**	2.42	1.50; 3.91 *	1.92	1.07; 3.45 *	1.47	0.94; 2.34	2.3	1.26; 4.18 *
**Age**	**<45 ^b^**	1		1		1		1	
	**45–54**	1.42	0.86; 2.38	0.50	0.27; 0.91 *	1.23	0.72; 2.08	0.81	0.40; 1.62
	**>54**	1.63	0.81; 3.27	0.69	0.30; 1.59	1.97	1.07; 3.65 *	0.69	0.33; 1.45
**Children**	**No ^b^**	1		1		1		1	
	**Yes**	0.67	0.38; 1.17	0.55	0.27; 1.12	0.93	0.55; 1.58	0.78	0.38; 1.60
**Bank**	**Local ^b^**	1		1		1		1	
	**National**	0.94	0.55; 1.61	1.93	1.07; 3.48 *	1.85	1.17; 2.93 *	1.5	0.83; 2.71
**Commercial role**	**No ^b^**	1		1		1		1	
	**Yes**	0.9	0.52; 1.58	1.02	0.53; 1.97	1.14	0.66; 1.96	0.89	0.45; 1.75
**Job position**	**Employee ^b^**	1		1		1		1	
	**Manager**	1.03	0.55; 1.94	1.38	0.68; 2.83	0.81	0.46; 1.40	0.98	0.47; 2.08
**Smoker**	**No ^b^**	1		1		1		1	
	**Yes**	1.7	0.89; 3.22	0.72	0.35; 1.49	0.93	0.52; 1.96	0.77	0.37; 1.59
**Drugs ^a^**	**No ^b^**	1		1		1		1	
	**Yes**	1.09	0.61; 1.95	3.77	1.51; 9.42 *	2.00	1.16; 3.46 *	0.52	0.26; 1.04
**Positivity Scale**		1.04	0.98; 1.10	1.01	0.94; 1.09	0.88	0.83; 0.93 *	0.92	0.85; 0.99 *
**Job Demand**		1.01	0.96; 1.05	1.11	1.05; 1.17 *	1.07	1.02; 1.11 *	1.10	1.04; 1.16 *
**Decision Latitude**		0.99	0.97; 1.02	0.96	0.93; 0.99 *	0.98	0.95; 1.01	0.95	0.92; 0.99 *
**Hosmer-Lemeshow’s Test**		0.364		0.827		0.290		0.413	

* the significant association between the item of BEST8 and socio-professional characteristics with JDCMs, *p* < 0.05; ^a^ Previous or present consumptions of antidepressants or sedatives drugs; ^b^ Reference group for OR.

**Table 4:** Multivariate logistic regression models of the last four BEST8′ items.

Covariates		Frequent Company’s Re-Organization Make Me Feel Uncomfortable		The Requests of Sales and/or Consultations Are in Conflict with What I Consider Morally Right		I have Time to Dedicate Myself to My Hobbies/Activities/Stuff		My Colleagues or Superiors Ask Me to Be More Flexible with the Job	
		OR	95% CI	OR	95% CI	OR	95% CI	OR	95% CI
**Gender**	**Male ^b^**	1		1		1		1	
	**Female**	1.08	0.63; 1.84	2.31	1.38; 3.87 *	0.58	0.37; 0.90 *	1.72	1.10; 2.70 *
**Age**	**<45 ^b^**	1		1		1		1	
	**45–54**	0.81	0.48; 1.39	0.78	0.50; 3.87	1.30	0.81; 2.11	0.89	0.52; 1.53
	**>54**	0.88	0.41; 1.88	0.68	0.30; 1.58	1.23	0.60; 2.52	0.78	0.42; 1.44
**Sons**	**No ^b^**	1		1		1		1	
	**Yes**	0.86	0.47; 1.56	1.51	0.85; 2.69	0.48	0.29; 0.79 *	0.85	0.50; 1.44
**Bank**	**Local ^b^**	1		1		1		1	
	**National**	1.51	0.90; 2.55	4.24	2.45; 7.34 *	1.97	1.22; 3.18 *	1.25	0.79; 1.99
**Commercial role**	**No ^b^**	1		1		1		1	
	**Yes**	1.05	0.90; 2.55	0.42	0.22; 0.79 *	1.27	0.75; 2.16	1.01	0.59; 1.72
**Job position**	**Employee ^b^**	1		1		1		1	
	**Manager**	1.11	0.57; 2.16	0.65	0.36; 1.17	0.73	0.43; 1.25	1.18	0.69; 2.00
**Smoker**	**No ^b^**	1		1		1		1	
	**Yes**	1.26	0.64; 2.48	0.69	0.36; 1.31	1.80	1.01; 3.20 *	0.81	0.46; 1.42
**Drugs ^a^**	**No ^b^**	1		1		1		1	
	**Yes**	1.61	0.83; 3.12	1.19	0.64; 2.22	0.59	0.34; 0.99	1.27	0.74; 2.19
**Positivity scale**		0.95	0.92; 0.98 *	0.90	0.84; 0.96 *	1.06	1.01; 1.13 *	1.01	0.94; 1.07
**Job demand**		1.09	1.04; 1.14 *	1.08	1.03; 1.13 *	0.89	0.85; 0.93 *	1.18	1.07; 1.16 *
**Decision Latitude**		0.95	0.92; 0.98 *	0.94	0.91; 0.97 *	0.99	0.97; 1.03	0.95	0.93; 0.98 *
**Hosmer-Lemeshow’s Test**		0.232		0.821		0.190		0.970	

* The significant association between the item of BEST8 and socio-professional characteristics with JDCMs, *p* < 0.05; ^a^ Previous or present consumptions of antidepressants or sedatives drugs; ^b^ Reference group.

## Supplementary Materials

The following are available online at http://www.mdpi.com/1660-4601/15/4/707/s1, Table S1: Univariate analysis of the 1st question of the BEST8, Table S2: Univariate analysis of the 2nd question of the BEST8, Table S3: Univariate analysis of the 3rd question of the BEST8, Table S4: Univariate analysis of the 4th question of the BEST8, Table S5: Univariate analysis of the 5th question of the BEST8, Table S6: Univariate analysis of the 6th question of the BEST8, Table S7: Univariate analysis of the 7th question of the BEST8, Table S8: Univariate analysis of the 8th question of the BEST8.
