# Supplementary material for: Are Bank Employees Stressed? Job Perception and Positivity in the Banking Sector: An Italian Observational Study
Source: Int J Environ Res Public Health. 2018 Apr 10;15(4):707. doi: 10.3390/ijerph15040707 (PMC5923749; doi:10.3390/ijerph15040707)
Supplement: Supplementary file 1 [file ijerph-15-00707-s001.pdf]

International Journal of
Environmental Research and Public Health

MDPI

Supplementary Materials: Are Bank Employees Stressed? Job Perception and Positivity in the Banking Sector: An Italian Observational Study

Alice Mannocci [1,*], Laura Marchini [2], Alfredo Scognamiglio [3], Alessandra Sinopoli [1], Simone De Sio [4], Sabina Sernia [5] and Giuseppe La Torre [1]

Table S1. Univariate analysis of the 1st question of the BEST8.

In terms of safety, It makes me uncomfortable thinking about a possible robbery on my desk								
Qualitative variables		I don't agree *		Agree		P [a]	OR	95% CI inf; sup
		N	%Row	N	%Row			
Gender	Male *	58	33	116	67	**<0.001**	**2.34**	**1.45;3.76**
	Female	37	18	173	82			
Age	<45 *	47	28	123	72	0.269	nc	nc
	45–54	29	20	115	80			
	>54	19	27	51	73			
Sons	NO *	23	20	90	80	0.198	0.71	0.42;1.20
	Yes	72	27	199	73			
Bank	Local *	37	25	111	75	0.925	1.02	0.63;1.65
	National	58	25	178	75			
Commercial role	NO *	28	24	87	76	0.907	0.97	0.58;1.61
	Yes	67	25	202	75			
Job position	Employee *	70	25	216	75	0.838	0.95	0.56;1.61
	Manager	25	26	73	74			
Smoker	NO *	81	27	225	73	0.119	1.65	0.88;3.10
	Yes	14	18	64	82			
Drugs [e]	NO *	71	26	205	74	0.475	1.21	0.72;2.06
	Yes	24	22	84	78			
Quantitative variables		mean	SD	mean	SD	P [b]		
Positivity Scale		23.8	3.4	24.2	4.4	0.474 [c]		
Job Demand		37.6	6.4	38	5.6	0.542 [d]		
Decision Latitude		62,0	10	62,0	8.7	0.995 [d]		

a : p-value χ^2 test; b: p-value t-student test for independent samples; c: equal Variances not assumed; d: equal Variances; e: Previous or present consumptions of antidepressants or sedatives drugs; n.c. the computation is not possible; * Reference group for OR; Bold: $p < 0.05$.

Table S2. Univariate analysis of the 2nd question of the BEST8.

The failure to achieve the budgets targets causes me anxiety, because there are risks of geographical mobility and/or of the switch of duties.								
Qualitative variables		I don't agree *		Agree		P [a]	OR	95% CI
		N	%	N	%			
Gender	Male *	40	23	134	77	**0.020**	**1.86**	**1.10;3.16**
	Female	29	14	181	86			
Age	<45 *	22	32	148	44	0.072	nc	nc
	45-54	32	22	112	78			
	>54	15	21	55	79			
Sons	NO *	14	12	99	88	0.066	0.55	0.30;1.05
	Yes	55	20	216	80			
Bank	Local *	37	25	111	75	**0.004**	**2.13**	**1.26;3.60**
	National	32	14	204	86			
Commercial role	NO *	26	23	89	77	0.122	1.53	0.89;2.65
	Yes	43	16	226	84			
Job position	Employee *	49	17	237	83	0.466	0.81	0.45;1.44
	Manager	20	20	78	80			
Smoker	NO *	55	18	251	82	0.996	1.00	0.52;1.91
	Yes	14	18	64	82			
Drugs [e]	NO *	62	23	214	77	**<0.001**	**4.18**	**1.85;9.46**
	Yes	7	7	101	93			
Quantitative variables		mean	SD	mean	SD	P [b]		
Positivity Scale		23.9	4.3	24.7	3.4	0.146 [d]		
Job Demand		38.6	5.7	35.0	5.5	**<0.001** [d]		
Decision Latitude		61.4	8.8	64.9	9.2	**0.004** [d]		

a: p-value χ^2 test; b: p-value t-student test for independent samples; c: equal Variances not assumed; d: equal Variances; e*:* Previous or present consumptions of antidepressants or sedatives drugs; n.c. the computation is not possible; * Reference group for OR; Bold: $p < 0.05$.

Table S3. Univariate analysis of the 3rd question of the BEST8.

The pace of change on work place exceeds my capacity for adaptation.								
Qualitative variables		I don't agree *		Agree		P [a]	OR	95% CI inf; sup
		N	%	N	%			
Gender	Male *	83	47.7	91	52	**0.023**	**1.61**	**1.07;2.42**
	Female	76	36.2	134	63.8			
Age	<45 *	78	45.9	92	54.1	0.166	nc	nc
	45–54	58	40.3	86	59.7			
	>54	23	32.9	47	61.1			
Sons	NO *	47	41.6	66	58.4	0.962	1.01	0.65;1.58
	Yes	112	41.3	159	58.7			
Bank	Local *	76	51.4	72	48.6	**0.002**	**1.94**	**1.28;2.96**
	National	83	35.2	153	64.8			
Commercial role	NO *	51	44.3	64	55.7	0.444	1.19	0.76;1.85
	Yes	108	40.1	161	59.9			
Job position	Employee *	116	40.6	170	59.4	0.565	0.87	0.55;1.39
	Manager	43	43.9	55	56.1			
Smoker	NO *	127	41.5	179	58.5	0.939	1.02	0.62;1.69
	Yes	32	41	46	59			
Drugs [e]	NO *	132	47.8	144	52.2	**<0.001**	**2.75**	**1.68;4.51**
	Yes	27	25	81	75			
Quantitative variables		Media (N)	SD	Media (N)	SD	P [b]		
Positivity Scale		25.5 (159)	4.1	23.1 (225)	3.9	**<0.001** [d]		
Job Demand		36.5(158)	5.3	39.0 (224)	6.0	**<0.001** [c]		
Decision Latitude		64.0 (159)	9.0	60.6 (225)	8.7	**<0.001** [d]		

a : p-value χ^2 test; b: p-value t-student test for independent samples; c: equal variances not assumed; d: equal Variances; e: Previous or present consumptions of antidepressants or sedatives drugs; n.c. the computation is not possible; * Reference group for OR; Bold: $p < 0.05$.

Table S4. Univariate analysis of the 4th question of the BEST8.

I'm not comfortable recommending a bank product just because in the budget.									
Qualitative variables		I don't agree *		Agree		P [a]	OR	95%CI Inf; sup
		N	%	N	%			
Gender	Male *	38	21.8	136	78.2	**0.006**	**2.17**	**1.24;3.78**
	Female	24	11.4	186	88.6			
Age	<45 *	25	14.7	145	85.3	0.410	nc	nc
	45-54	22	15.3	122	84.7			
	>54	15	21.4	55	78.6			
Sons	NO *	17	15	96	85	0.705	0.89	0.49;1.63
	Yes	45	16.6	226	83.4			
Bank	Local *	33	22.3	115	77.7	**0.009**	**2.05**	**1.18;3.55**
	National	29	12.3	207	87.7			
Commercial role	NO *	20	17.4	95	82.6	0.665	1.14	0.64;2.04
	Yes	42	15.6	227	84.4			
Job position	Employee *	42	14.7	224	85.3	0.184	0.67	0.37;1.21
	Manager	20	20.4	78	79.6			
Smoker	NO *	48	15.7	258	84.3	0.628	0.85	0.44;1.64
	Yes	14	17.9	64	82.1			
Drugs [e]	NO *	44	15.9	232	84.1	0.862	0.95	0.52;1.73
	Yes	18	16.7	90	83.3			
Quantitative variables		Mean (N)	SD	Mean (N)	SD	P [b]		
Positivity Scale		25.7 (62)	4.05	23.7(322)	4.11	**<0.001** [d]		
Job Demand		0.82 (61)	0.16	0.96 (321)	0.21	**<0.001** [c]		
Decision Latitude		65.4 (62)	8.84	61.4 (322)	8.8	**0.001** [d]		

a: p-value χ^2 test; b: p-value t-student test for independent samples; c: equal Variances not assumed; d: equal Variances; e: Previous or present consumptions of antidepressants or sedatives drugs; n.c. the computation is not possible; * Reference group for OR; Bold: $p < 0.05$.

Table S5. Univariate analysis of the 5th question of the BEST8

Frequent Company's re-organization make me feel uncomfortable								
Variabili qualitative		I don't agree *		Agree		P [a]	OR	95% CI inf;sup
		N	%	N	%			
Gender	Male *	41	23.6	133	76.4	0.466	1.20	0.74;1.94
	Female	43	20.5	167	79.5			
Age	<45 *	35	20.6	135	79.4	0.863	nc	nc
	45–54	33	22.9	111	77.1			
	>54	16	22.9	54	77.1			
Sons	NO *	22	19.5	91	80.5	0.461	0.82	0.47;1.41
	Yes	62	22.9	209	77.1			
Bank	Local *	43	29.1	105	70.9	**0.007**	**1.95**	**1.19;3.18**
	National	41	17.4	195	82.6			
Commercial role	NO *	29	25.2	86	74.8	0.300	1.31	0.78;2.20
	Yes	55	20.4	214	79.6			
Job position	Employee *	61	21.3	225	78.7	0.658	0.88	0.51;1.53
	Manager	23	23.5	75	76.5			
Smoker	NO *	70	22.9	236	77.1	0.347	1.36	0.72;2.56
	Yes	14	17.9	64	82.1			
Drugs [e]	NO *	69	25	207	75	**0.018**	**2.07**	**1.12;3.80**
	Yes	15	13.9	93	86.1			
Quantitative variables		Mean (N)	SD	Mean (N)	SD	P [b]		
Positivity		25.7 (62)	4.1	23.7 (322)	4.1	**0.001** [d]		
Job Demand		35.4(61)	5.3	38.4 (321)	5.3	**<0.001** [d]		
Decision Latitude		65.5 (62)	8.4	61.3 (322)	8.9	**<0.001** [d]		

a : p-value χ^2 test; b: p-value t-student test for independent samples; c: equal Variances not assumed; d: equal Variances; e: Previous or present consumptions of antidepressants or sedatives drugs; n.c. the computation is not possible; * Reference group for OR; Bold: $p < 0.05$.

Table S6. Univariate analysis of the 6th question of the BEST8.

The requests of sales and/or consultations are in conflict with what I consider morally right.										
Qualitative variables		No *		Yes		I don't know		P [a]	OR	95% CI inf; sup
		N	%	N	%	N	%			
Gender	Male *	69	39.7	94	54	11	6.3	**0.002**	**2.01**	**1.30;3.12**
	Female	54	25.7	148	70.5	8	3.8			
Age	<45 *	52	30.6	109	64.1	9	5.3	0.875	Nc	nc
	45–54	48	33.3	91	63.2	5	3.5			
	>54	23	32.9	42	60	5	7.1			
Sons	NO *	42	37.2	67	59.3	4	3.5	0.202	1.35	0.85;2.16
	Yes	81	29.9	175	64	15	5.5			
Bank	Local *	71	48	66	44.6	11	7.4	**<0.001**	**3.64**	**2.31;5.75**
	National	52	22	176	74.6	8	3.4			
Commercial role	NO *	28	24.3	72	62.6	15	13	0.16	0.70	0.42;1.51
	Yes	95	35.3	170	63.2	19	4.9			
Job position	Employee *	82	28.7	189	66.1	15	5.2	**0.018**	**0.56**	**0.35;0.91**
	Manager	41	41.8	53	54.1	4	4.1			
Smoker	NO *	93	30.4	198	64.7	15	4.9	0.163	0.69	0.41;1.17
	Yes	30	38.5	44	56.4	4	5.1			
Drugs [e]	NO *	96	34.8	165	59.8	15	5.4	**0.048**	**1.66**	**1.00;2.75**
	Yes	27	25	77	71.3	4	3.7			
Quantitative variables		Mean (N)	SD	Mean (N)	SD	Mean (N)	SD	P [b]		
Positivity Scale		25.7 (123)	3.8	23.2 (242)	4.1	23.7 (19)	4.2	**<0.001** [d]		
Job Demand		36.6 (122)	5.2	38.9 (241)	5.9	34.0 (19)	5.7	**<0.001** [d]		
Decision Latitude		64.5 (123)	8.2	60.0 (242)	8.8	65.8 (19)	8.9	**<0.001** [d]		

a: p-value χ^2 test, the "I don't know" data was not included in the analysis. b: p-value t-student test for independent samples, the "I don't know" data was not included in the analysis. c: equal Variances not assumed; d: equal Variances; e: Previous or present consumptions of antidepressants or sedatives drugs; n.c. the computation is not possible; * Reference group for OR; Bold: $p < 0.05$.

Table S7. Univariate analysis of the 7th question of the BEST8.

I have time to dedicate myself to my hobbies/activities/stuff										
Qualitative variables		No*		Yes		I don't know		P [a]	OR	95% CI inf; sup
		N	% Riga	N	% Riga	N	% Riga			
Gender	Male *	78	44.8	95	54.6	1	0.6	**0.001**	**0.51**	**0.34;0.77**
	Female	128	61	80	38	2	1			
Age	<45 *	90	52.9	77	45.3	3	1.8	0.849	nc	nc
	45–54	80	55.6	64	44.4	0	0			
	>54	36	51.4	34	48.6	0	0			
Sons	NO*	49	43.4	63	55.8	1	0.9	**0.009**	**0.56**	**0.36;0.87**
	Yes	157	57.9	112	41.3	2	0.7			
Bank	Local *	85	57.4	61	41.2	2	1.4	0.200	1.31	0.87;1.99
	National	121	51.3	114	48.3	1	0.4			
Commercial role	NO *	63	54.8	52	45.2	0	0	0.854	1.04	0.67;1.62
	Yes	142	53.2	123	45.7	3	1.1			
Job position	Employee *	147	51.4	136	47.6	3	1	0.157	0.71	0.45;1.14
	Manager	59	60.2	39	39.8	0	0			
Smoker	NO *	169	55.2	134	43.8	3	1	0.187	1.40	0.85;2.30
	Yes	37	47.4	41	52.6	0	0			
Drugs[e]	NO *	133	48.2	141	51.1	2	0.7	**0.001**	**0.44**	**0.27;0.70**
	Yes	73	67.6	34	31.5	1	0.9			
Quantitative variables		Mean (N)	SD	Mean (N)	SD	Mean (N)	SD	P [b]		
**Positivity Scale**		23.4 (206)	4.1	24.9 (175)	4.1	20.7 (3)	4.5	**0.001** [d]		
**Job Demand**		39.5 (205)	5.6	36.0 (174)	5.6	39.7 (3)	5.1	**<0.001** [d]		
**Decision Latitude**		61.9 (206)	8.9	62.1 (175)	9.2	58.7 (3)	9.5	0.832 [d]		

a: p-value χ^2 test, the "I don't know" data was not included in the analysis. b: p-value t-student test for independent samples, the "I don't know" data was not included in the analysis. c: equal Variances not assumed; d: equal Variances; e: Previous or present consumptions of antidepressants or sedatives drugs; n.c. the computation is not possible; * Reference group for OR; Bold: $p < 0.05$.

Table S8. Univariate analysis of the 8th question of the BEST8.

My colleagues or superiors ask me to be more flexible with the job.										
Qualitative variables		No *		Yes		I don't know		P [a]	OR	95% CI inf; sup
		N	%	N	%	N	%			
Gender	Male *	74	42.5	98	56.3	2	1.1	**0.016**	**1.67**	**1.10;2.55**
	Female	65	31	144	68.6	1	0.5			
Age	<45 *	58	34.1	110	64.7	2	1.2	0.273	nc	nc
	45-54	50	34.7	94	65.3	0	0			
	>54	31	44.3	38	54.3	1	1.4			
Sons	NO *	39	34.5	73	64.6	1	0.9	0.664	0.90	0.57;1.43
	Yes	100	36.9	169	62.4	2	0.7			
Bank	Local *	64	43.2	82	55.4	2	1.4	**0.019**	**1.67**	**1.09;2.55**
	National	75	31.8	82	55.4	1	0.4			
Commercial role	NO *	48	41.7	66	57.4	1	0.9	0.136	1.41	0.90;2.21
	Yes	91	33.8	176	65.4	2	0.7			
Job position	Employee *	103	36	180	62.9	3	1	0.952	0.99	0.61;1.59
	Manager	36	36.7	62	63.3	0	0			
Smoker	NO *	110	35.9	194	63.4	2	0.7	0.810	0.94	0.56;1.57
	Yes	29	37.2	48	61.5	1	1.3			
Drugs [e]	NO *	110	39.9	166	60.1	0	0	**0.027**	**1.74**	**1.06;2.84**
	Yes	29	26.9	76	70.4	3	2.8			
Quantitative variables		Mean N)	SD	Mean (N)	**SD**	**Mean (N)**	**SD**	**P [b]**		
Positivity Scale		24.6 (139)	4.0	23.8 (242)	4.3	23.8(3)	4.3	0.063 [d]		
Job Demand		35.8 (137)	5.8	39.2 (242)	5.5	34.7(3)	7.8	**<0.001 [d]**		
Decision Latitude		64.2 (139)	8.6	60.8 (242)	9.0	56.7 (3)	6.4	**<0.001 [d]**		

a: p-value χ^2 test, the "I don't know" data was not included in the analysis. b: p-value t-student test for independent samples, the "I don't know" data was not included in the analysis. c: equal Variances not assumed; d: equal Variances; e: Previous or present consumptions of antidepressants or sedatives drugs; n.c. the computation is not possible; * Reference group for OR; Bold: $p < 0.05$.

© 2017 by the authors. Submitted for possible open access publication under the terms and conditions of the Creative Commons Attribution (CC BY) license (http://creativecommons.org/licenses/by/4.0/).
